# Atmospheric Vapor Impact on Desert Vegetation and Desert Ecohydrological System

**DOI:** 10.3390/plants12020223

**Published:** 2023-01-04

**Authors:** Zhiming Xin, Wei Feng, Hongbin Zhan, Xuying Bai, Wenbin Yang, Yiben Cheng, Xiuqin Wu

**Affiliations:** 1 School of Soil and Water Conservation, Beijing Forestry University, Beijing 100083, China; 2 The Sand Forestry Experimental Center, Chinese Academy of Forestry, Hohhot 015200, China; 3 Department of Grass and Livestock, Xilingol Vocational College, Xilingol League 026000, China; 4 Department of Geology and Geophysics, Texas A&M University, College Station, TX 77843, USA; 5 Low-Coverage Sand Control Company, Hohhot 010000, China

**Keywords:** Ulan Buhl Desert, arid area, tamarisk, reverse sap flow, atmospheric vapor

## Abstract

The ability of plants to absorb unsaturated atmospheric water vapor is a controversial topic. To study how vegetation in arid areas survives under limited water resources, this study uses Tamarisk in the Ulan Buh Desert of China as an example. The in-situ observation of a newly designed Lysimeter and sap flow meter system were used to monitor the precipitation infiltration and the utilization efficiency of Tamarisk of atmospheric vapor. The results show that the annual precipitation of 84 mm in arid areas could still result in deep soil recharge (DSR) with a recharge rate of 5 mm/year. Furthermore, DSR is detectable even in the winter, and the 5-year average DSR was 5.77% of the annual precipitation. It appears that the small precipitation events are critically important for the survival of Tamarisk. When the atmospheric relative humidity reaches 70%, Tamarisk leaves can absorb the unsaturated atmospheric vapor, which accounts for 13.2% of the annual precipitation amount. To adapt to the arid environment, Tamarisk can harvest its water supply from several sources including atmospheric vapor and micro-precipitation events (whose precipitation is below the measurement limit of 0.2 mm of the precipitation gauge) and can still permit a certain amount of recharge to replenish the deep soil moisture. Such an ecohydrological dynamic is of great significance to desert vegetation.

## 1. Introduction

Water restricts the growth of plants in almost all terrestrial ecosystems [1], and desert vegetation is more sensitive to the impact of water shortage [2]. Long-term water shortage would affect the function of grassland ecosystems [3], especially the desert grasslands in Inner Mongolia [4]. The amount of precipitation (Pr) in these regions is small, and it is difficult to form surface runoff; thus, the precipitated water rarely flows into rivers or transforms into deep soil recharge (DSR) or groundwater [5,6]. It is an indisputable fact that atmospheric vapor can be absorbed by desert vegetation [7]. For instance, researchers have tracked the process of atmospheric vapor replenishing leaf using H-O isotopes, dyes, and other tracers, but it is difficult to quantify the amount of unsaturated atmosphere vapor absorbed by the leaves [8,9].

There are many sources of atmospheric vapor, including micro-precipitation in arid areas [10], where micro-precipitation refers to precipitation below the measurement limit of the precipitation gauge of 0.2 mm. The common type of precipitation in desert areas is micro-precipitation, which is unstable and difficult to observe with conventional rain gauges [11,12]. This kind of micro-precipitation is the main source of atmospheric vapor in the desert [13,14]. The significance of these micro-precipitations to the ecosystem in the arid area is still an open question. Some researchers argue that micro-precipitation is invalid precipitation in the sense that it is evaporated into the atmosphere even before reaching vegetation and soil, thus cannot provide water resources for arid ecosystems [15]. Some other researchers state that micro-precipitation plays an important role in recharging soil and groundwater in the arid ecosystem [16]. For instance, studies have found that the amount of atmospheric condensate could reach 5.0% to 16.2% of the annual precipitation in the Negev Arid in Israel, the Nevada Desert in the United States, and the Atacama Desert in Chile [17,18,19]; thus, atmospheric vapor is an important water source for the ecosystems in those arid and semi-arid regions [20,21]. The condensed water converted from atmospheric vapor recharges soil moisture and also directly improves the physiological status of plant leaves [22]. Some studies report that night dew could change the water shortage of Ponderosa pine (Pinus ponderosa) and extend its survival time under drought stress [23,24]. Research results in the Namib Desert of Africa show that desert plants can absorb the dew attached to the surface of leaves [25].

What is already known is that vegetation in arid areas is sensitive to rainfall, fog, and condensation. What is unknown is the atmospheric vapor effect on vegetation in arid areas [26,27]. It is generally difficult to quantify the water absorbed by the leaves; thus, it is unclear whether the water absorbed by leaves has any physiological effect on the plants or not. With the rapid development of technologies such as the thermal diffusion flow method (Sap flow meter) and leaf water potential measurement, recent studies have confirmed that in forest ecosystems, tropical precipitation forest ecosystems, and desert ecosystems, some plants can absorb condensed water on their leaves [28]. Unsaturated atmospheric vapor and condensed atmospheric water can enter the leaves through leaf hairs and cuticles and migrate to the xylem through the mesophyll tissue [29]. However, related research on this subject only describes this physiological process in a qualitative manner without providing rigorous quantitative analysis, which is crucial for assessing the role played by the water absorbed by the leaves in the overall physiological function of ecosystems.

Based on the above analysis, the two processes of deep sandy soil recharge (or DSR) via precipitation-induced infiltration in desert areas and the ability of vegetation to absorb atmospheric vapor through leaves have been confirmed. To resolve the issues, we set up a field experiment to study the recharge effect of precipitation on deep soil under the in-situ state of Ulan Buh Desert and to examine whether Tamarisk leaves show any sign of absorption and utilization of atmospheric vapor. Based on the experimental results, we will try to answer four questions: (1) Can precipitation in desert areas recharge deep soil or groundwater? (2) Does the Tamarisk in the Ulan Buh desert have the ability to absorb atmospheric vapor? (3) What is the proper boundary condition for the leaves to absorb atmospheric vapor? (4) How efficiently do the leaves absorb atmospheric vapor? (5) What are the percentages of atmospheric vapor absorption through leaves of the annual precipitation?

## 2. Research Area

**Figure 1:**
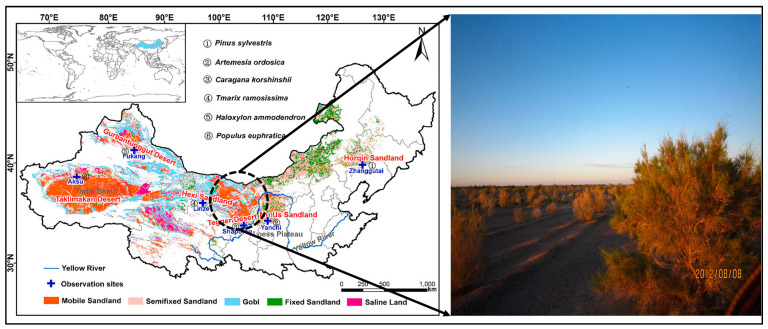
Location of the experimental area and research objects. The figure on the left is the distribution map of China’s deserts and sandy land; different vegetations were selected to be planted in these areas to combat desertification [32]. The experimental site is located in the transition zone of the arid desert zone to semi-arid sandy land. The picture on the right is the Tamarisk forest in Ulan Buh Desert.

The research area is located at the northeastern edge of the Ulan Buh Desert (106°00′–107°20′ E, 39°40′–41°00′ N) of China, with an average elevation of 1050 m above mean sea level, as shown in Figure 1. The research area is flat and the soil type is mainly fine sand, and it has a semi-arid continental climate with an average annual precipitation of 98 mm, an average annual temperature of 6.8 °C, and an annual sunshine duration of 3229.9 h. The groundwater level is 9 m below the ground surface [26,30]. The main type of plants in the area is native Tamarisk Ramosissima (Tamarisk), and the main types of other plantations are *Haloxylon ammodendron*, *Hedysarum scoparium*, and *Caragana korshinskii*. Natural herbaceous vegetation mainly includes *Artemisia ordosica*, *Nitraria tangutorum*, and so on. The experimental site is located in an artificial Tamarisk Ramosissima forest with relatively flat terrain and small undulation [31]. The Tamarisk forest is about 30 years old and is planted with a row space of 3 m × 2 m. The average base diameter of Tamarisk is 9.34 cm, the average height of Tamarisk is 2.95 m, and the average crown width of Tamarisk is 2.69 m × 2.32 m. After excavation, we find that the deepest root depth of native plants is about 6 m, but most of the roots of artificial vegetation are concentrated at 0–1.5 m depths.

## 3. Materials and Methods

### 3.1. Deep Soil Recharge Observation

**Figure 2:**
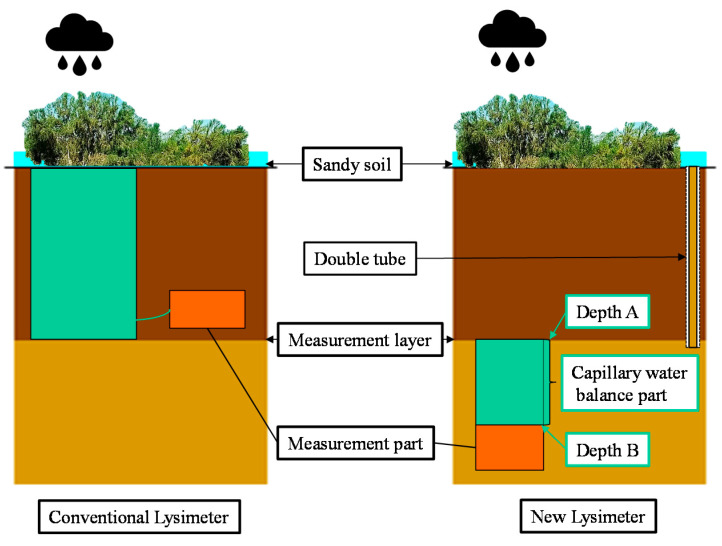
Schematic diagram of the recharge process of DSR observed via the newly designed Lysimeter. Left is the traditional Lysimeter; right is the newly designed Lysimeter. (The conventional Lysimeter has a massive semi-closed container in which plants are planted inside; the newly designed Lysimeter has a small shape, and the upper interface of this instrument is the measuring surface, which can be set in any depth of soil without the need for vegetation planted inside).

To monitor DSR, a newly designed Lysimeter is used in this research [26,33]. This new Lysimeter is assembled with two parts: a balance part and a measurement part. As shown in Figure 2, the function of the balance part is to ensure that the soil moisture infiltration into this part can be completely transported to the downward measurement part. The water balance part uses a cylindrical with an impervious side wall to wrap the undisturbed in-site soil column. The length of the soil column is determined based on the local soil particle size and the capillary water rising (which is less than 50 cm for the fine sandy soil of the site). The advantage of this design is that when the soil at depth B (in Figure 2) reaches the saturated state, the capillary rise can at most reach depth A (see Figure 2); thus, the soil moisture cannot overflow from the top of the soil column at depth A. When there is soil moisture infiltrating into the water balance part at depth A, the soil moisture at depth B discharges into the underneath measuring part. The measuring part calculates the amount of water discharged from the upper balance part via a weighing (or precipitation) gauge.

When installing the newly designed Lysimeter in sandy land, we need to do our best not to damage the root system and the soil structure of the rhizosphere. The sandy soil layer often collapses because of the loose structure. Before installing the Lysimeter, we need to irrigate the sandy land in the plot regularly to prevent soil collapse. After irrigation, we use an excavator to excavate a section of 3.2 m depth and then manually excavate 0.3 m laterally (toward the main root of the Tamarisk) to install the Lysimeter, which is 0.3 m length, 0.3 m width, and 1.2 m height into the excavated space. This installation method enables two features: (1) measuring the amount of DSR below the Tamarisk root system and (2) not damaging the root system and soil structure of the Tamarisk. The excavation revealed that there is no obvious soil layering at the site. We used in-situ sandy soil for backfilling and the backfill sand was continuously irrigated to make sure each soil layer was relatively compact.

### 3.2. Sap Flow Observation

#### 3.2.1. In-Situ Observation

In this experiment, 4 Tamarisk plants were selected as experimental plants; the tree diameter and height were chosen to roughly represent the stand structure at the site. We use SF-3 HPV sap flow monitoring system (East 30 Sensor, Pullman, WA, USA); this special sensor has a central heater needle and two thermistors needle up and down [34]. The sensor can be used in thin stems as small as 1 cm in diameter. A sap flow sensor was installed on the north-facing side. We wrapped the probe with foam soft plastic first and then wrapped it further with reflective plastic paper and injected glue at both sides. The data are collected with CR-300 (Campbell Sci, Logan, UT, USA) with an interval of 6 min. The diameter of Tamarisk is measured via a vernier caliper. Sap velocities are calculated according to Equation (1) [35,36]:

(1) $$V_{sap}=\frac{2k}{C_{w}\left(r_{u}+r_{d}\right)}\ln\left(\frac{\Delta T_{u}}{\Delta T_{d}}\right)$$

where *k* is the sapwood thermal connectivity, set to 0.5 Wm^−1^K^−1^, $C_{w}$ is the special heat capacity of water, *r* is the distance of heating needle to the measuring needle, ΔT is the temperature difference before and after the heating, and *u* and *d* stand for location up and down of the heater sensor.

We correct the sap velocity as the bark is damaged during the drilling process and affects the sap flow results [37]. Based on the corrected sap velocity and the sapwood area at the monitor point, we can calculate the sap flow; then, we can selectively use sap velocity and sap flow [38]. The correction equation and sap flow calculation equations are as follows:

(2) $$V_{c}=bV_{sap}+cV_{sap}^{2}+dV_{sap}^{3}$$

(3) $$A_{sap}=\pi {\left(d-d_{bark}\right)}^{2}-\pi {\left(d-d_{bark}-d_{sap}\right)}^{2}$$

(4) $$F=V_{C}*A_{sap}$$

where $V_{c}$ (cm·h^−1^) is the corrected $V_{sap}$, and *b*, *c*, and *d* are the correction coefficients. We set *b* = 1.8558, *c* = −0.0018 h·cm^−1^, *d* = 0.0003 h^2^·cm^−2^ according to the literature [35,39]. $A_{sap}$ is sapwood area (cm^2^), calculated using the power law function, d is measuring point diameter, $d_{bark}$ is bark thickness, $d_{sap}$ is sapwood thickness, and *F* is sap flux (cm^3^·h^−1^).

#### 3.2.2. Determination of the Tipping Point of the Leaf Absorption of Atmospheric Vapor

**Figure 3:**
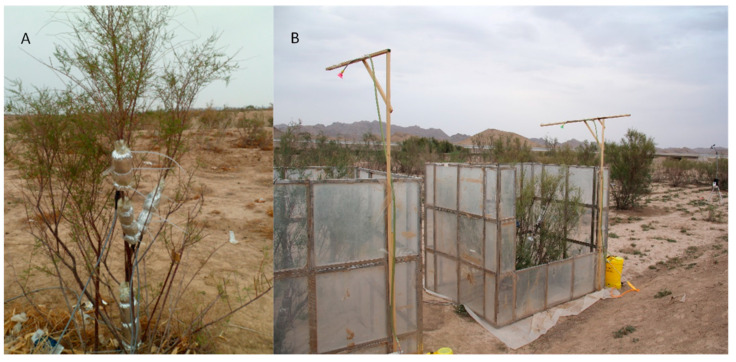
In-site observation settings of the experimental observation field ((**A**) was the Tamarisk in the in-situ state, and (**B**) was the Tamarisk in the humidified control room).

When the relative humidity (RH) of the air reaches a certain level, Tamarisk leaves start to absorb atmospheric vapor, and such a condition is named the critical condition in this study. As shown in Figure 3, we designed an in-situ control room which can simulate humidification of Tamarisk under in-situ conditions. By controlling the RH of the atmosphere, we can determine the critical condition for Tamarisk to absorb atmospheric vapor. As shown in Figure 3, a plastic film is laid on the lower part of the control room (ground surface) to prevent water from infiltrating into the soil layer during humidification.

#### 3.2.3. Calculation of Leaf Water Absorption

The atmospheric vapor absorbed by the leaves includes the moisture stored in the leaves absorbed by the leaves and the moisture transported downward through the sap flow after absorption by the leaves (the so-called reverse sap flow). To measure the atmospheric vapor absorbed by the leaves and stored in the leaves, we selected 20 Tamarisk plants with different growth statuses in the experimental plot and divided them into two groups (ten plants per group), one group with humidification and one group without humidification. From May to September of 2020, we continuously monitored the sap flow for a week per month and measured the atmospheric vapor absorption and reverse sap flow of Tamarisk under humidified conditions. After the experiment, all the Tamarisk branches were cut off, and the Tamarisk leaves on the branches were collected and brought back to the laboratory for drying. The dry matter was weighed and the water absorption of the leaves after humidification was accurately calculated. Comparing the difference between the moisture content of the leaves in the humidified and non-humidified groups allows the amount of atmospheric vapor that has been stored in leaves per unit dry mass to be deduced. To convert the water absorption of Tamarisk on a single plant scale to the water absorption of Tamarisk per unit crown size, we cut down the Tamarisk after the experiment and collected all the leaves, which were dried and weighed, and when combined with the crown width, one can use Equation (1) to calculate the absorption of atmospheric vapor by the Tamarisk per unit area, Equation (2) to calculate the total sap flux of a plant based on the dry mass of the whole plant, where DLM is the dry leaf mass and 0.0573 is obtained through the linear regression of observed sap flux and DLM (details are presented in Figure 3A).

### 3.3. Air Relative Humidity Observation and Simulation

**Figure 4:**
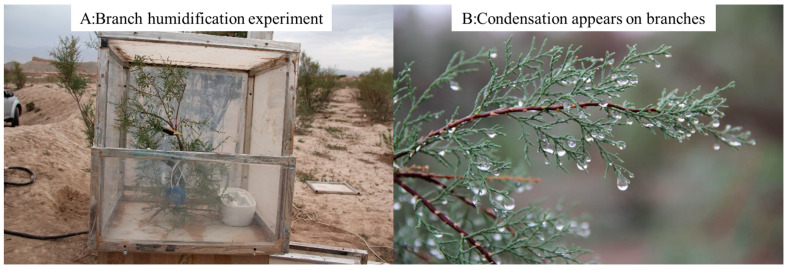
(**A**) Control room for humidifying parts of the Tamarisk branches, collection of wetted leaves, and counting of the difference in water content between water-absorbing and non-water-absorbing leaves. (**B**) Condensation on tamarisk branches observed under high RH conditions.

At a certain relative humidity, Tamarisk is able to absorb atmospheric vapor, and therefore, we can estimate how much water will be absorbed from the atmosphere during the year based on the meteorological conditions (Figure 4B). To facilitate the computation, we have established a HOBO H21 small automatic weather station on the experimental site to record precipitation, atmospheric relative humidity, and other environmental information. The precipitation sensor is a precipitation gauge (S-RGB-M002, METER, Pullman, WA, USA), and the air temperature and humidity sensor are S-THB-M002 (Onset, Logan, UT, USA).

There is another question that we tried to answer: if the RH remains above the critical level, will the rate of atmospheric vapor absorbed by leaves change with time or not? To answer this question, we specifically designed an experiment, as shown in Figure 4A. In this experiment, we set up a small control room and changed the RH level for a branch of Tamarisk only. We selected 20 Tamarisk trees and humidified half of the Tamarisk branches of those 20 trees. When the reverse sap flow appeared, we cut down both the humidified Tamarisk branches and the non-humidified Tamarisk branches, counted the leaves, and obtained the weight of the humidified and non-humidified Tamarisk leaves.

## 4. Results

### 4.1. Water Balance and Deep Soil Recharge Characteristics

**Figure 5:**
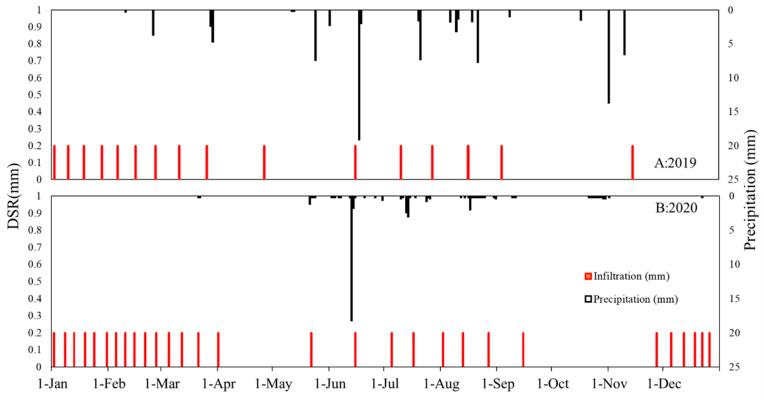
Annual precipitation and DSR variations in 2019 and 2020.

The precipitation in the experimental site is concentrated from May to August, but the DSR in May to August is relatively small, which was not entirely anticipated at first. This is probably due to the strong evapotranspiration in the summer months (May–August), thus leading to relatively small DSR to recharge the deep soil and groundwater. It turns out that the main form of winter precipitation is snowfall, and the amount of DSR during the winter months is greater than the DSR in the summer months, as shown in Figure 5. This is not entirely surprising to us, as in the winter, the shallow soil layer is sometimes frozen (up to a depth of 1 m); thus, the evaporation has been substantially reduced. On the other hand, the frozen shallow soil will also substantially reduce the downward infiltration from rain or snow to replenish the deep soil. Consequently, the source of water for DSR most likely comes from condensed water from the soil water vapor underneath the frozen soil layer. As a result, the DSR throughout the year is mostly concentrated in December–April. As shown in Table 1, during the five-year experimental period, the DSR accounts for 5.77% of the precipitation over the same period.

In Table 1, the annual precipitation of the experimental site in 2020 is 84 mm, and the DSR of the same year is 5 mm. The five-year average DSR accounts for 5.77% of the precipitation in the same year. The evapotranspiration of the experimental site is strong during the summer months, making it difficult for shallow sandy soil to retain soil moisture during the summer months. Consequently, a large amount of precipitation returns to the atmosphere through strong evapotranspiration, and a small part of it infiltrates into the deep soil layer and groundwater.

### 4.2. Critical Conditions for Reverse Sap Flow

**Figure 6:**
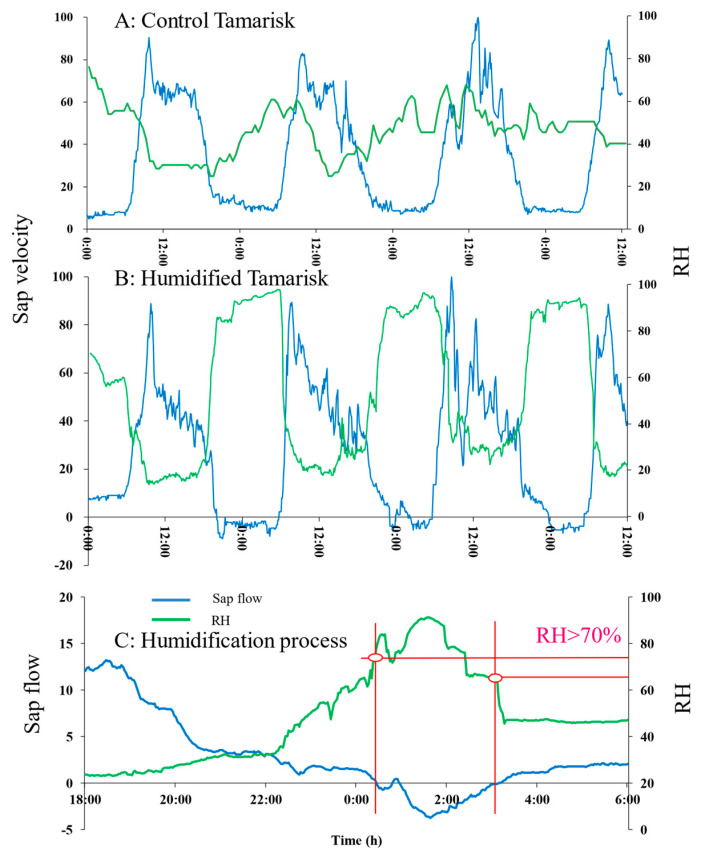
Time of occurrence of reverse sap flow. (**A**) The sap flow characteristics of Tamarisk in the in-situ state, (**B**) the sap flow characteristics of Tamarisk under humidified conditions. (**C**) The critical condition of reversed sap flow and corresponding RH, humidification process and dehumidification process.

We humidify the Tamarisk during the nighttime, starting at 18:00 each day to 12:00 of the next day for 18 h. Studies have shown that even at night, leaf stomata do not close, and nocturnal transpiration happens [40,41]. Our experiment plot has the highest temperature at about 14:00, so we had to stop the experiment at around that time to ensure that Tamarisk in the control room would not die due to the high temperature. A humidification experiment was carried out for three consecutive days, to verify that Tamarisk in the control room can continue to maintain the state of absorbing atmospheric vapor. Unfortunately, Tamarisk 1 died eventually, probably because it had stayed in the control room for too long. The stem sap flow data indicate that Tamarisk leaves begin to absorb and utilize atmospheric vapor when the air humidity exceeds 80%, as shown in Figure 6. Specifically, when we started to humidify the air to make the RH greater than 80%, we observed obvious reverse sap flow, and we observed no reverse flow during the same period for the case without humidification. As shown in Figure 6A–C, three consecutive days of experiments showed that reverse sap flow appears after humidification. This experimental result suggests that the Tamarisk leaves can indeed absorb atmospheric vapor, and the critical condition for atmospheric vapor absorption is met when the relative humidity reaches 80%.

A notable point is that such a critical condition is determined at the moment when the reverse sap flow starts to appear, RH > 70%. The actual critical condition for the Tamarisk leaves starting to absorb atmospheric vapor might be lower than this value because the leaves may start to absorb some atmospheric vapor for their own use at the beginning stage of absorption without transmitting the adsorbed water down to the stem. At present, it is unclear whether the leaves would preferentially absorb atmospheric vapor for their own use or transmit it to the stem. In the future, we need to find a reliable method of describing these two competitive processes (storage of water in the leaves and transmission of water from leaves to stem).

### 4.3. The Relationship between the Sap Flow and the Dry Matter Quality of the Leaves

The results are shown in Table 2. The average amount of atmospheric vapor absorbed by the leaves per unit dry mass is 0.955 g/g. In other words, each gram of dry leaves matter would absorb 0.955 g of atmospheric vapor. Therefore, when we use the sap flow meter to monitor the absorption of atmospheric vapor by Tamarisk leaves, this portion of atmospheric vapor amount should be counted.

**Figure 7:**
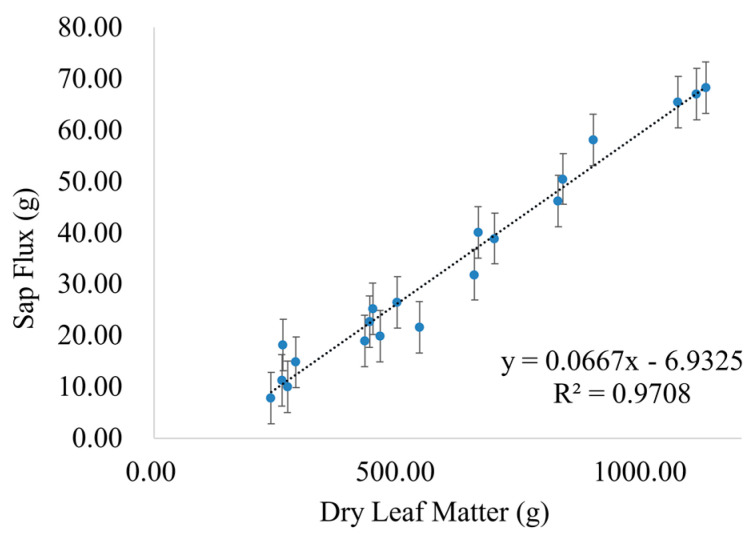
The relationship between sap flux and dry leaf mass.

According to the sap flux and leaf dry matter data, as shown in Table 2, we can establish the relationship between the dry leaf matter mass and the sap flow flux (as shown in Figure 7), which appears to be positively correlated, and the relationship is shown in Equation (2). Based on this equation, we can estimate the amount of atmospheric vapor absorption of the Tamarisk by measuring the dry leaf matter mass (DLM) of the Tamarisk.

### 4.4. The Relationship between the Liquid Flow Rate and the Duration of High Relative Humidity

**Figure 8:**
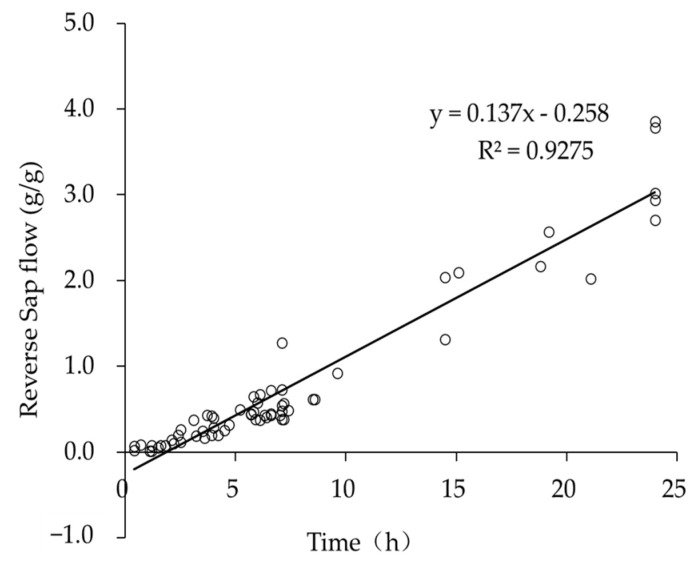
The relationship between the reverse flow and the duration of the high relative humidity during the growing season from May to August in 2020.

There are several questions of concern here. The first question is: Do Tamarisk leaves continuously absorb atmospheric vapor under conditions of high relative humidity? The second question is: What is the threshold for leaves to absorb atmospheric vapor? To answer these two questions, we conducted 60 observation experiments of high relative humidity duration and sap flow amount in the in-situ state. Specifically, we placed Tamarisk in a control room with high air relative humidity and measured the direction of sap flow of Tamarisk (from the stem to the leaf is the positive sap flow, and from the leaf to the stem is the reverse sap flow). The experiment was carried out during the growing season from May to August in 2020, and the results are shown in Figure 8.

One should note that the water vapor absorption capacity of Tamarisk leaves in different seasons is different. The amount of water vapor absorption of leaves at the beginning of the growing season is relatively weak, and the amount of atmospheric vapor absorbed by leaves can be ignored, but the amount of water vapor absorption in the mature period is strong and leaves will absorb more atmospheric vapor. The atmospheric vapor absorption rate under different water stresses will also show differences. The budding period of Tamarisk is relatively short (in two weeks). In our experiment, the crown width of Tamarisk and the final collected Tamarisk leaves are at their mature-dormant state. To avoid the statistically significant differences in the results of Tamarisk absorption of atmospheric vapor, we distributed these 60 observations event evenly throughout the growing season (12 observations per month). The advantage of this is that the water absorption bias caused by the difference in the amount of Tamarisk leaves in different seasons can be avoided; thus, the Tamarisk water absorption model accuracy can be guaranteed. Arid areas rarely have conditions under which the high relative humidity duration exceeds 24 h, so most of the observation time is less than 10 h.

As shown in Figure 6, the reverse flow mount is a function of the duration of the relative humidity (RH > 80%). Using the relationship between reverse sap flow and dry leave mass weight, combined with the change of leaf content before and after humidification, a water vapor absorption estimation model based on unit dry leave mass weight is built. The relationship between the weight of the dry leaf matter and the sap flow amount is shown in Equation (5). We weighed the atmospheric vapor absorption of 20 branches and the results show that Tamarisk leaves begin to absorb atmospheric vapor before the sap flow is formed. The average atmospheric vapor absorption amount is 0.955 g/g. Therefore, based on the amount of atmospheric vapor absorbed and stored in the unit leaf (Equation (5)) and the sap flow transmitted to the stem, we established an atmospheric vapor absorption model (Equation (6)).

*W* = 0.1173*t* (5)

*W* = *w* + 0.955 (6)

where *W* is the total water absorption per unit leaf dry weight (g/g), *w* is the water delivery per unit leaf dry weight (g/g), and *t* is time (h); the constant 0.955 in Equation (6) is the water storage per unit leaf dry weight (g/g).

**Figure 9:**
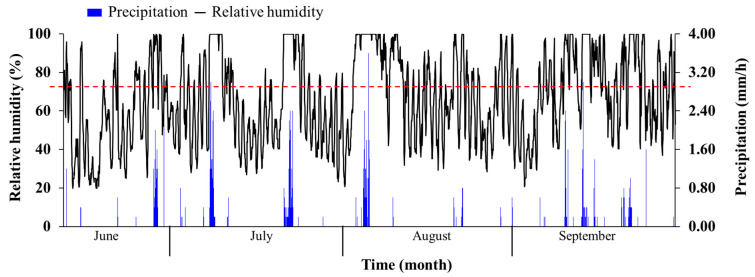
The distribution of relative humidity in the growing season of Tamarisk, 2020.

Meteorological data are shown in Figure 9 and the absorption of atmospheric vapor model result is shown in Table 3. The precipitation duration from May to September of 2020 during the growing season is 91 h (the relative humidity during the precipitation period is 100%), during which the leaves absorbed 11.6293 g/g of atmospheric vapor. The duration for the high relative humidity of the atmosphere (RH > 80%) is 870 h. During this period, leaves absorbed 103.006 g/g atmospheric vapor. If we only want to count the amount of atmospheric vapor absorbed by the leaves, we have to minus the precipitation duration time, then the amount of water absorbed by the leaves from the unsaturated atmosphere is 91.4307 g/g. Based on the model built in Section 3.1, we can calculate the distribution characteristics of precipitation in the Tamarisk forest land in 2020 and the amount of atmospheric vapor absorbed by the Tamarisk. As shown in Figure 5 and Table 1 and 3, the precipitation and DSR in 2020 was 84 mm and 5 mm, respectively, and the leaves absorbed 11.088 mm atmospheric vapor per unit area. The atmospheric vapor absorbed by the leaves during the growing season accounted for 13.2% of the annual precipitation in 2020.

## 5. Discussion

We conducted experiments on water sources of Tamarisk in arid areas on two scales (plot scale and individual plant scale) and quantified the redistribution process of precipitation in the plot and the critical conditions for Tamarisk to absorb atmospheric vapor. To accomplish the objectives, we used a newly designed Lysimeter to monitor the redistribution process of precipitation in sandy land for five years. The special porous structure of the sandy land allows the moderate precipitation in the arid area to recharge the deep soil and groundwater, in the areas with significant potential evaporation. The traditional view is that precipitation in arid areas has no effect on deep soil recharge [42], and precipitation quickly returns to the atmosphere after it reaches the land surface [43,44]. Our research finds that 5.77% of the precipitation is capable of infiltrating into the deep soil.

Although it is difficult to estimate the amount of atmospheric vapor absorbed by Tamarisk, we are still able to use the weighing method to carry out repeated experiments to measure the contribution of atmospheric vapor absorbed by Tamarisk relatively accurately. Garnier’s modified thermal diffusion sap flow meter [26,44,45] has become the widely used monitoring method in the study of transpiration and has been applied by many scholars around the world. This study has confirmed this conclusion by using the new sap flow meter to monitor sap flow in both directions and calculate the amount of atmospheric vapor absorbed by Tamarisk. Researchers have analyzed the relationship between Tamarisk sap flow flux and meteorological factors and found that Tamarisk sap flow also exists at night [46,47]. We have further confirmed this point and observe that when the relative humidity of atmospheric vapor is higher than 80%, Tamarisk can absorb atmospheric vapor.

The main meteorological element that affected Tamarisk sap flow was the air–water pressure difference and air temperature [48]. A study on the water consumption characteristics of Tamarisk in the Gurbantunggut Desert showed that different soil conditions have different influences on the sap flow flux of Tamarisk [49]. This study, however, has found that atmospheric vapor absorbed by Tamarisk can be transported to the stem/trunk, but whether this moisture can enter the root system or even enter the soil layer is still unclear, and further research is needed. The study of Tamarisk sap flow in the shelter forest in the Taklimakan Desert found that the stem sap flow has a positive correlation with solar radiation, air temperature, and wind speed but has a negative correlation with relative humidity [50]. This suggests that not only the relative humidity affects the absorption of atmospheric vapor by Tamarisk, but other factors may also affect the absorption of atmospheric vapor by Tamarisk. Possibly under certain conditions, even if the relative humidity is less than 80%, Tamarisk can absorb atmospheric vapor. To verify such a hypothesis, we need to conduct long-term, large-scale observation.

There are several limitations that are briefly summarized as follows. Firstly, part of the leaf surface shows condensation when the relative humidity of the air is higher than 80% but under 100%. At this time, the leaves not only absorb atmospheric vapor, but they can also cause condensation of water on the surface of leaves, which is not specifically quantified. Secondly, the accuracy of the rain gauge used for monitoring precipitation is 0.2 mm, and the so-called micro precipitation events (with precipitation less than 0.2 mm) are not recorded using this instrument. Therefore, it is likely that the precipitation may be underestimated due to it not counting the micro-precipitation events. Thirdly, the branches of Tamarisk may also absorb some vapor, which is not counted in this study. Fourthly, this study does not investigate how the atmospheric vapor absorbed by the leaves participates in the physiological process of vegetation. It also does not answer the question of whether, after the leaves absorb atmospheric vapor, the absorbed water is preferentially transmitted to the stem or used by leaves. We have focused mostly on the factor of atmospheric vapor in this study, and further attention should be paid to the influences of other parameters in the future.

## 6. Conclusions

This research has monitored the distribution process of precipitation in the Tamarisk Forest land in arid areas through in-situ measurements, especially the measurement of deep soil recharge. Tamarisk is an excellent drought and salinity tolerant plant, absorbing water vapor from the atmosphere under specific conditions to maintain survival. We have gained great insights on the characteristics of Tamarisk utilization of atmospheric vapor and established a model to quantify the water vapor absorption process of Tamarisk. The main conclusions are as follows.

(1) Precipitation in arid areas can recharge the deep soil layer, and the atmosphere–vegetation–soil continuum still maintains hydrological connectivity. The five-year average DSR accounted for 5.77% of the mean annual precipitation.
(2) Atmospheric vapor in desert areas is an important water source for native vegetation. The condition for Tamarisk leaves to absorb atmosphere vapor is that the relative humidity reaches more than 70%. Micro-precipitation is also an important water source for native desert vegetation.
(3) Atmospheric vapor enters Tamarisk leaves in two forms: part of the vapor is stored in the leaves with a strength of 0.955 g/g and part of the vapor is transported downwards in the form of reverse sap flow.
(4) The efficiency of Tamarisk absorbing atmospheric vapor through the leaves is 102.263 g/g, accounting for 13.2% of the annual precipitation. Atmospheric vapor provides one of the water resources necessary for the survival of Tamarisk in semi-arid regions.

## Figures and Tables

**Table 1:** Monthly cumulative precipitation—DSR amount in every month from 2016 to 2020.

Month	Precipitation (mm)	DSR (mm)	D/P
January	0	3.4	5.77%
February	0.6	3	
March	4.2	2	
April	33.2	0.6	
May	23.8	1.2	
June	79.4	1	
July	40.6	1.2	
August	92	1.8	
September	18.6	0.6	
October	25.8	0.2	
November	3.8	0.6	
December	0.2	3	

**Table 2:** Sampling statistics of dry mass weight and sap flow of Tamarisk, 2020.

Sample No.	Branch Diameter (mm)	Sap Flux (g)	Leaf Dry Matter (g)	Unhumidified Leaves Weight (g)	Humidification Leaves Weight (g)	Net Absorption/Dry Matter (g/g)	Average Leaf Storage (g/g)
1	15.320	1108.669	67.014	172.272	238.456	0.988	0.955
2	13.250	496.674	26.431	67.946	95.476	1.042	
3	10.930	695.551	38.902	100.005	140.936	1.052	
4	12.640	661.792	40.128	103.157	140.314	0.926	
5	7.330	262.880	18.191	46.763	65.001	1.003	
6	9.890	655.135	31.852	81.882	113.557	0.994	
7	13.374	440.367	22.651	58.229	80.112	0.966	
8	10.130	541.867	21.586	55.491	74.093	0.862	
9	10.410	238.431	7.840	20.154	27.886	0.986	
10	9.960	289.750	14.794	38.031	52.327	0.966	
11	6.925	260.725	11.218	28.838	38.336	0.847	
12	5.319	272.088	10.046	25.825	35.417	0.955	
13	11.900	826.030	46.235	118.856	160.275	0.896	
14	13.400	897.730	58.153	149.494	204.689	0.949	
15	12.331	447.028	25.208	64.802	90.112	1.004	
16	12.500	429.829	18.995	48.830	65.869	0.897	
17	13.200	461.180	19.943	51.267	70.265	0.953	
18	18.347	1071.258	65.419	168.172	230.665	0.955	
19	17.760	1127.940	68.251	175.452	240.172	0.948	
20	15.620	835.645	50.494	129.805	175.336	0.902	

**Table 3:** Precipitation, growing season, and high humidity duration in 2020.

**Environmental Information**	**Pr (mm)**	**DSR (mm)**	**Pr Time (h)**	**RH > 70% Duration (h)**	**Growing Season (h)**	**Vapor Absorption/Canopy Size (m^2^)**	**Average Absorption/Pr (mm/mm)**
	84	5	91	870	3600	6.456	/
Vapor absorption	/	/	11.629	103.006	/	/	13.20%

## Data Availability

All the data are available from the corresponding author on reasonable request.
